# Crystal structure of 6-azido-6-de­oxy-1,2-*O*-iso­propyl­idene-α-d-gluco­furan­ose

**DOI:** 10.1107/S2056989020012438

**Published:** 2020-09-18

**Authors:** Adam Wood, Paul V. Bernhardt, Ian van Altena, Michela I. Simone

**Affiliations:** a Discipline of Chemistry, University of Newcastle, Callaghan, NSW 2308, Australia; b Priority Research Centre for Drug Development, University of Newcastle, Callaghan, NSW 2308, Australia; c School of Chemistry and Molecular Biosciences, University of Queensland, Brisbane 4072, Australia

**Keywords:** crystal structure, imino­sugar, d-glucose, tosyl­ation, azide, regioselectivity, glycosidase inhibition

## Abstract

Short syntheses to high *Fsp* ^3^ index natural-product analogues such as imino­sugars are of paramount importance in the investigation of their biological activities and reducing the use of protecting groups is an advantageous synthetic strategy. An iso­propyl­idene group was employed towards the synthesis of seven-membered ring imino­sugars and the title compound, C_9_H_15_N_3_O_5_, was crystallized as an inter­mediate, in which the THF ring is twisted and the dioxolane ring adopts an envelope conformation: the dihedral angle between the rings is 67.50 (13)°. In the crystal, the hydroxyl groups participate in O—H⋯(O,O) and O—H⋯N hydrogen-bonding inter­actions, which generate chains of mol­ecules propagating parallel to the *a-*axis direction. There is a notable non-classical C—H⋯O hydrogen bond, which cross-links the [100] chains into (001) sheets.

## Abstract 2

Short syntheses to high *Fsp* ^3^ index natural-product analogues such as imino­sugars are of paramount importance in the investigation of their biological activities and reducing the use of protecting groups is an advantageous synthetic strategy. In this case only an iso­propyl­idene group was employed towards the synthesis of seven-membered ring imino­sugars.

## Chemical context   

The installation of various functionalities *via N*- and/or *O*-alkyl­ation has been shown to impart improved biological profiles and potencies to imino­sugars (Šesták *et al.*, 2018 ▸; Prichard *et al.*, 2018 ▸; Simone *et al.*, 2012 ▸; Sayce *et al.*, 2016 ▸, Woodhouse *et al.*, 2008 ▸; Johnson & Houston, 2002 ▸). Diminishing the number of synthetic steps to the imino­sugar building blocks that are precursors to their alkyl­ated con­geners is advantageous. Many imino­sugar syntheses start from monosaccharide starting materials (Wood *et al.*, 2018 ▸; Lee *et al.*, 2012 ▸; Rasmussen & Jensen, 2011 ▸). Reducing the number of protecting groups and removing the need for purification by chromatography are useful strategies to a more expedited synthesis of analogues (Katritzky *et al.*, 1991 ▸; Steiner *et al.*, 2009 ▸; Liu *et al.*, 2014 ▸).
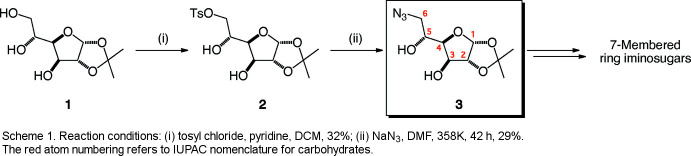


In the present study, the only protecting group that was used to synthesize seven-membered ring imino­sugars was an iso­propyl­idene group (acetonide) to make inter­mediate **1** from d-glucose. Selective tosyl­ation of the primary hydroxyl group, followed by nucleophilic displacement with sodium azide afforded the title compound **3**, C_9_H_15_N_3_O_5_ (Tsuchiya *et al.*, 1981 ▸; Fleet *et al.*, 1989 ▸), see Scheme 1 ▸.

Primary alcohols can be tosyl­ated regioselectively over secondary alcohols (Johnson *et al.*, 1963 ▸). There are examples of mono­tosyl­ation of monosaccharides and analogues using di-*n*-butyl­tin oxide and di­methyl­amino­pyridine as catalyst (Tsuda *et al.*, 1991 ▸) and of cyclo­dextrins (Yamamura & Fujita, 1991 ▸; Ashton *et al.*, 1991 ▸; Fujita *et al.*, 1992 ▸). Any mechanistic ambiguities that may have arisen from the S_N_2 reaction with azide ions was clarified by X-ray crystallographic analysis, which confirmed the structure of the title compound as described below.

## Structural commentary   

**Figure 1:**
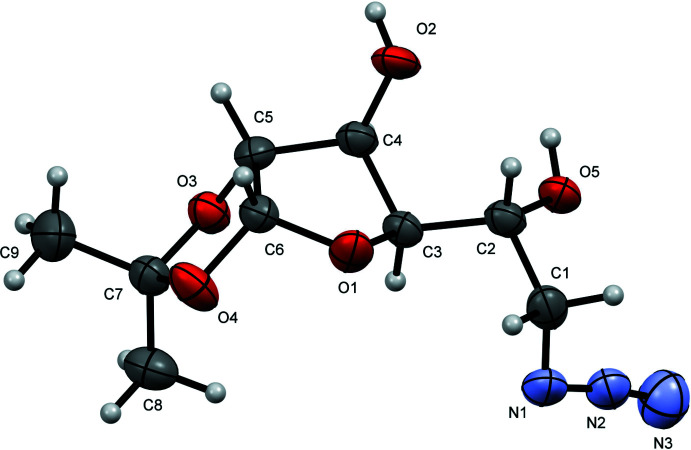
The mol­ecular structure of **3** showing 50% displacement ellipsoids.

In compound **3** (Fig. 1 ▸), the tetra­hydro­furan (THF) ring is best described as twisted with atoms C3 and C4 displaced by 0.169 (3) and −0.384 (2) Å, respectively, from the plane through C5/C6/O1. The fused dioxolane ring adopts an envelope conformation with O3 displaced by 0.402 (2) Å from the mean plane of the other ring atoms (C5/C6/O4/C7; r.m.s. deviation = 0.005 Å). The dihedral angle between the five-membered rings (all atoms) is 67.50 (13)°. The hydroxyl group O2—H2*A* and the acetonide oxygen atom O3 project axially from the THF ring, lying respectively above and below in a *trans* arrangement from one another [O2—C4—C5—O3 = 164.46 (18)°]. The other two groups projecting from the THF ring are O4 of the acetonide and the side chain attached to C3, which sit equatorially. The absolute structure of **3** was not definitively established in the refinement but the configurations of the stereogenic atoms (C2 *R*, C3 *R*, C4 *S*, C5 *R* and C6 *R*) were set to match those of the starting material.

## Supra­molecular features   

**Figure 2:**
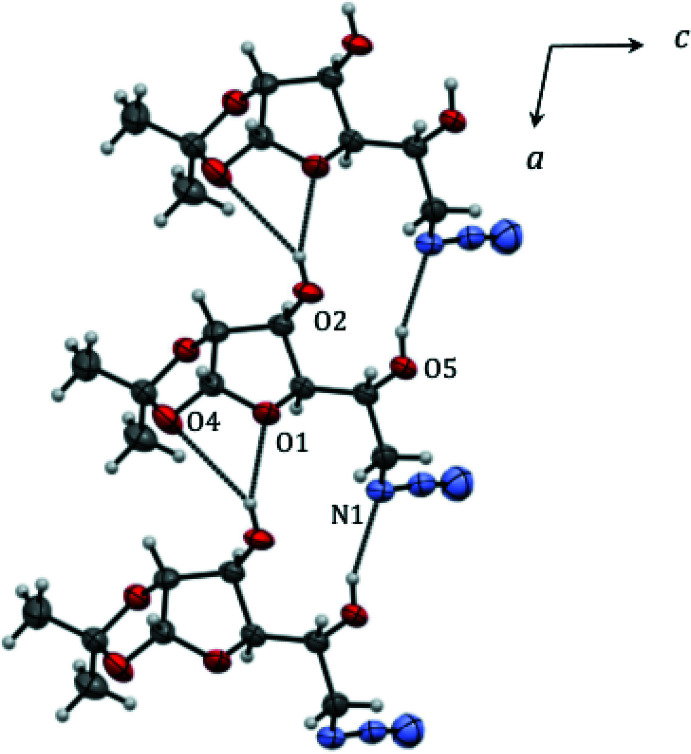
Partial packing diagram for **3** showing hydrogen bonds as dashed lines.

There are no intra­molecular hydrogen-bonding inter­actions in **3** but both hydroxyl groups participate in inter­molecular hydrogen-bonding inter­actions (Table 1 ▸, Fig. 2 ▸), which generate chains propagating parallel to the *a*-axis direction. The O5 hydroxyl group donates a hydrogen bond to the proximal azide N atom [O5—H5*A*⋯N1^i^; H⋯N = 2.12 Å; O—H⋯N = 157°; symmetry code: (i) *x* − 1, *y*, *z*]. The other group (O2) is involved in an asymmetric, bifurcated hydrogen-bond to the THF ring O atom (O2—H*2A*⋯O1^i^; 2.09 Å; 154°) and a weaker contact with one of the dioxolane O-atoms (O2—H2*A*⋯O4^i^; 2.71 Å; 150°). There is a notable non-classical hydrogen-bond [C6—H6⋯O5^ii^; 2.38 Å; 159°; symmetry code: (ii) −*x*, *y* − 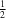, −*z* + 2], which cross-links the [100] chains into (001) sheets.

## Database survey   

**Figure 3:**
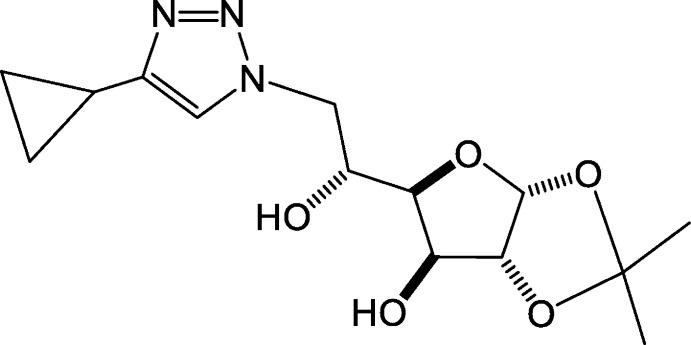
Structure of **4** (see text).

The most closely related crystal structure in the literature is **4** (Fig. 3 ▸), the 4-cyclo­propyl-1,2,3-triazole derivative of compound **3** [Zhang *et al.*, 2013 ▸, Cambridge Structural Database (Groom *et al.*, 2016 ▸) refcode NINQOS] synthesized from a copper-catalysed azide–alkyne cyclo­addition of the tribenzyl ether analogue of **3** followed by deprotection with NH_3_/NaOH (Pradere *et al.*, 2008 ▸). Conversion of the azide to a triazole removes the hydrogen-bonding capability of the proximal N atom and the packing in this structure is distinctly different with a hydrogen-bonded network being present. Other points of difference in structure **4** relative to **3** include the free hydroxyl group on the THF ring, which adopts an axial conformation, and the dioxolane ring methyl groups tilted closer to the THF ring.

Other examples of crystal structures of α-d-gluco­furan­ose derivatives constrained by a 1,2-*O*-iso­propyl­idene or analog­ous protecting group include: 3-*O*-ethyl-3-*C*-nitro­methyl-1,2;5,6-di-*O*-iso­propyl­idene-α-d-gluco­furan­ose (Ivanovs *et al.*, 2016 ▸; QENNEF) and 3-*O*-benzyl-1,2-*O*-iso­propyl­idene-5-*O*-methane­sulfonyl-6-*O*-triphenyl-methyl-α-d-gluco­furan­ose and its azide displacement product (Clarke *et al.*, 2018 ▸; QIBFUF). A general observation is that groups departing from O-3 take up axial or quasi-axial orientations relative to the THF ring in all cases examined and as is the case for the 3-*O*-ethyl group in QENNEF and the benzyl groups in QIBFUF and (4*R*)-4-carbamoyl-4-[(4*R*)-3-*O*-benzyl-1,2-*O*-iso­propyl­idene-β-l-threo­furanos-4-*C*-yl]-oxazolidin-2-one (Steiner *et al.*, 2009 ▸) and the tosyl­ate group in 1,2:5,6-di-*O*-iso­propyl­idene-3-*O*-toluene­sulfonyl-α-d-gluco­furan­ose (Mamat *et al.*, 2012 ▸). The impact of perfluorination on the conformation of monosaccharide derivatives was probed on (*R*/*S*)-*N*-benzyl-*N*-(5-de­oxy-1,2-*O*-iso­propyl­idene-3-*O*-methyl-α-d-xylo­furanos-5-yl)-2,3,3,3-tetra­fluoro­propanamide and analogous com­pounds (Bilska-Markowska *et al.*, 2017 ▸). The crystal structures of α-d-gluco­furan­ose-1,2:3,5-bis­(phen­yl)boronate and α-d-gluco­furan­ose-1,2:3,5-*bis*(*p*-tol­yl)boronate highlight modulation in structures according to a temperature gradient (Chandran & Nangia, 2006 ▸). The structure of chloro­(cyclo­penta­dien­yl)bis­(1,2:5,6-di-*O*-iso­propyl­idene-α-d-gluco­furan­os-3-*O*-yl)titanate provides insight into the use of monosaccharides as ligands in complexes. The titanium atom is bonded to two monosaccharide OH-3, in axial positions, a cyclo­penta­dienyl and a chloride ligand, to take up a three-legged piano stool arrangement (Riediker *et al.*, 1989 ▸). The unit cell of (*R*)-3-de­oxy-1,2:5,6-di-*O*-iso­propyl­idene-α-d-glu­co­furanos-3-yl-*tert*-butane­sulfinate contains four symmetry-independent mol­ecules with the *tert*-butyl and glucose moieties turned away from each other in order to minimize steric repulsion (Chelouan *et al.*, 2018 ▸).

## Synthesis and crystallization   

**1,2-** ***O*** **-Iso­propyl­idene-6-** ***O*** **-** ***p*** **-toluene­sulfonyl-α-d-gluco­furan­ose, 2:**

A solution of freshly recrystallized tosyl chloride (0.479 g, 2.55 mmol) in DCM (1.6 ml) was added dropwise over 20 min to a stirring solution of 1,2-*O*-iso­propyl­idene-α-d-gluco­furan­ose **1** (0.513 g, 2.32 mmol) in pyridine (3.8 ml) and DCM (4.2 ml), under an atmosphere of nitro­gen. The reaction was stirred at room temperature for 48 h. TLC analysis (EtOAc/cyclo­hexane 2:3) revealed the formation of one product (*R*_f_ = 0.45). After adding DCM (10 ml), the reaction mixture was washed with 1 *M* HCl (1 ml). The DCM layer was dried to give 1,2-*O*-iso­propyl­idene-6-*O*-*p*-toluene­sulfonyl-α-d-gluco­furan­ose **2** (0.282 g, 32%) as an off-white crystalline solid. δ_H_ (CDCl_3_, 400 MHz) 7.79 (2H, *d*, *J* = 8.3 Hz, 2 Ar-H), 7.35 (2H, *d*, *J* = 8.1 Hz, 2 Ar-H), 5.88 (1H, *d*, *J* = 3.6 Hz, H-1), 4.50 (1H, *d*, *J* = 3.6 Hz, H-2), 4.36 (1H, *d*, *J* = 2.7 Hz, H-3), 4.29 (1H, *dd*, *J* = 10.2, 2.5 Hz, H-6), 4.19 (1H, *td*, *J* = 7.7, 2.5 Hz, H-5), 4.11 (1H, *dd*, *J* = 10.2, 6.8 Hz, H-6′), 4.01 (1H, *dd*, *J* = 7.7, 2.7 Hz, H-4), 2.45 (3H, *s*, Ar—CH_3_), 1.45, 1.29 (6H, 2 *s*, 2 acetonide CH_3_). δ_C_ (acetone-*d*_6_, 100 MHz): 145.2 (ArCq—S), 132.3 (ArCq—CH_3_), 130.0 (2 ArC), 128.0 (2 ArC), 111.9 (Cq acetonide), 105.1 (C-1), 85.0 (C-2), 79.4 (C-4), 75.0 (C-3), 72.1 (C-6), 68.0 (C-5), 26.8 (acetonide CH_3_), 26.2 (acetonide CH_3_), 21.7 (Ar—CH_3_); ν_max_ (cm^−1^): 3426, 3322, 2979, 2928, 1378, 1215, 1162, 1058, 1037, 1007, 962, 883, 850, 673, 657, 626.

**6-Azido-6-de­oxy-1,2-** ***O*** **-iso­propyl­idene-α-d-gluco­furan­ose, 3:**

Sodium azide (0.290 g, 4.46 mmol) was added to a stirring solution of **2** (1.668 g, 4.45 mmol) in DMF (18 ml) at room temperature. The reaction mixture was then heated to 358 K for 42 h. TLC analysis (EtOAc/cyclo­hexane 2:3) revealed complete consumption of the starting material (*R*_f_ = 0.45) and the formation of one product (*R*_f_ = 0.30). The crude product was dried and successively dissolved in 1,4-dioxane with addition of hexane to yield an off-white precipitate, which was filtered off. The remaining filtrate contained 6-azido-6-de­oxy-1,2-*O*-iso­propyl­idene-α-d-gluco­furan­ose, **3**. Crystallization was achieved overnight at 248 K, after dissolution in diethyl ether with addition of hexane. The ether–hexane solution was recrystallized to obtain 2nd and 3rd crops of product to yield a combined 0.319 g (29%) of product **3** as a white crystalline solid. δ_H_ (CDCl_3_, 400 MHz): 5.95 (1H, *d*, *J* = 3.7 Hz, H-1), 4.53 (1H, *d*, *J* = 3.6 Hz, H-2), 4.37 (1H, *d*, *J* = 2.8 Hz, H-3), 4.16 (1H, *td*, *J* = 6.6, 3.6 Hz, H-5), 4.05 (1H, *dd*, *J* = 6.6, 2.8 Hz, H-4), 3.61 (1H, *dd*, *J* = 12.7, 3.5 Hz, H-6), 3.55 (1H, *dd*, *J* = 12.7, 6.5 Hz, H-6′), 1.49, 1.32 (6H, 2 *s*, 2 acetonide CH_3_). ν_max_ (cm^−1^): 3442, 2992, 2938, 2109, 1385, 1376, 1215, 1164, 1066, 1048, 1008, 955, 881, 854, 788, 674.

## Refinement   

Crystal data, data collection and structure refinement details are summarized in Table 2 ▸. All H atoms were positioned geometrically (O—H = 0.84, C—H = 0.98–1.00 Å) and refined as riding with *U*_iso_(H) = 1.2*U*_eq_(O,C) or 1.5*U*_eq_(C-methyl).

## Supplementary Material

Crystal structure: contains datablock(s) global, I. DOI: 10.1107/S2056989020012438/hb7940sup1.cif

Structure factors: contains datablock(s) I. DOI: 10.1107/S2056989020012438/hb7940Isup2.hkl

CCDC reference: 1968033

Additional supporting information:  crystallographic information; 3D view; checkCIF report

## Figures and Tables

**Table 1:** Hydrogen-bond geometry (Å, °)

*D*—H⋯*A*	*D*—H	H⋯*A*	*D*⋯*A*	*D*—H⋯*A*
O2—H2*A*⋯O1^i^	0.84	2.09	2.871 (2)	154
O2—H2*A*⋯O4^i^	0.84	2.71	3.462 (2)	150
O5—H5*A*⋯N1^i^	0.84	2.12	2.910 (3)	157
C6—H6⋯O5^ii^	1.00	2.38	3.332 (3)	159

Symmetry codes: (i) 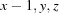; (ii) 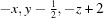.

**Table 2:** Experimental details

Crystal data	
Chemical formula	C_9_H_15_N_3_O_5_
*M*_r_	245.24
Crystal system, space group	Monoclinic, *P*2_1_
Temperature (K)	190
*a*, *b*, *c* (Å)	5.7615 (4), 9.7752 (8), 10.6833 (9)
β (°)	101.255 (8)
*V* (Å^3^)	590.11 (8)
*Z*	2
Radiation type	Cu *K*α
μ (mm^−1^)	0.97
Crystal size (mm)	0.40 × 0.30 × 0.02

Data collection	
Diffractometer	Rigaku Xcalibur, EosS2, Gemini ultra
Absorption correction	Multi-scan (*CrysAlis PRO*; Rigaku, 2015 ▸)
*T*_min_, *T*_max_	0.741, 1
No. of measured, independent and observed [*I* > 2σ(*I*)] reflections	3705, 1788, 1674
*R*_int_	0.044
θ_max_ (°)	61.5
(sin θ/λ)_max_ (Å^−1^)	0.570

Refinement	
*R*[*F* ^2^ > 2σ(*F* ^2^)], *wR*(*F* ^2^), *S*	0.036, 0.086, 1.08
No. of reflections	1788
No. of parameters	156
No. of restraints	1
H-atom treatment	H-atom parameters constrained
Δρ_max_, Δρ_min_ (e Å^−3^)	0.13, −0.15
Absolute structure	Flack (1983 ▸)
Absolute structure parameter	−0.4 (3)

Computer programs: *CrysAlis PRO* (Rigaku, 2015 ▸), *SIR92* (Altomare *et al.*, 1994 ▸), *SHELXL97* (Sheldrick, 2008 ▸), *ORTEP-3 for Windows* and *WinGX* publication routines (Farrugia, 2012 ▸).

## supplementary crystallographic information

### Crystal data

**Table:**

C_9_H_15_N_3_O_5_	*F*(000) = 260
*M**_r_* = 245.24	*D*_x_ = 1.38 Mg m^−^^3^
Monoclinic, *P*2_1_	Cu *K*α radiation, λ = 1.54184 Å
Hall symbol: P 2yb	Cell parameters from 1783 reflections
*a* = 5.7615 (4) Å	θ = 6.2–60.6°
*b* = 9.7752 (8) Å	µ = 0.97 mm^−^^1^
*c* = 10.6833 (9) Å	*T* = 190 K
β = 101.255 (8)°	Plate, colourless
*V* = 590.11 (8) Å^3^	0.40 × 0.30 × 0.02 mm
*Z* = 2	

### Data collection

**Table:**

Rigaku Xcalibur, EosS2, Gemini ultra diffractometer	1788 independent reflections
Radiation source: fine-focus sealed X-ray tube	1674 reflections with *I* > 2σ(*I*)
Graphite monochromator	*R*_int_ = 0.044
Detector resolution: 8.0217 pixels mm^-1^	θ_max_ = 61.5°, θ_min_ = 4.2°
ω scans	*h* = −6→6
Absorption correction: multi-scan (CrysAlisPro; Rigaku, 2015)	*k* = −11→10
*T*_min_ = 0.741, *T*_max_ = 1	*l* = −12→12
3705 measured reflections	

### Refinement

**Table:**

Refinement on *F*^2^	Secondary atom site location: difference Fourier map
Least-squares matrix: full	Hydrogen site location: inferred from neighbouring sites
*R*[*F*^2^ > 2σ(*F*^2^)] = 0.036	H-atom parameters constrained
*wR*(*F*^2^) = 0.086	*w* = 1/[σ^2^(*F*_o_^2^) + (0.0346*P*)^2^ + 0.0212*P*] where *P* = (*F*_o_^2^ + 2*F*_c_^2^)/3
*S* = 1.08	(Δ/σ)_max_ < 0.001
1788 reflections	Δρ_max_ = 0.13 e Å^−^^3^
156 parameters	Δρ_min_ = −0.14 e Å^−^^3^
1 restraint	Absolute structure: Flack (1983)
Primary atom site location: structure-invariant direct methods	Absolute structure parameter: −0.4 (3)

### Special details

**Table:**

Geometry. All s.u.'s (except the s.u. in the dihedral angle between two l.s. planes) are estimated using the full covariance matrix. The cell s.u.'s are taken into account individually in the estimation of s.u.'s in distances, angles and torsion angles; correlations between s.u.'s in cell parameters are only used when they are defined by crystal symmetry. An approximate (isotropic) treatment of cell s.u.'s is used for estimating s.u.'s involving l.s. planes.
Refinement. Refinement of F^2^ against ALL reflections. The weighted R-factor wR and goodness of fit S are based on F^2^, conventional R-factors R are based on F, with F set to zero for negative F^2^. The threshold expression of F^2^ > 2σ(F^2^) is used only for calculating R-factors(gt) etc. and is not relevant to the choice of reflections for refinement. R-factors based on F^2^ are statistically about twice as large as those based on F, and R- factors based on ALL data will be even larger.

### Fractional atomic coordinates and isotropic or equivalent isotropic displacement parameters (Å^2^)

**Table:**

	*x*	*y*	*z*	*U*_iso_*/*U*_eq_	
C1	0.3329 (4)	0.6279 (3)	1.2308 (2)	0.0428 (6)	
H1A	0.3365	0.5953	1.3189	0.051*	
H1B	0.4098	0.5574	1.1863	0.051*	
C2	0.0786 (4)	0.6435 (2)	1.1633 (2)	0.0345 (5)	
H2	−0.0043	0.5539	1.1654	0.041*	
C3	0.0532 (4)	0.6893 (3)	1.0256 (2)	0.0329 (5)	
H3	0.1366	0.7784	1.0212	0.04*	
C4	−0.2022 (4)	0.6991 (2)	0.9528 (2)	0.0340 (5)	
H4	−0.2721	0.7914	0.9614	0.041*	
C5	−0.1750 (4)	0.6702 (2)	0.8164 (2)	0.0342 (5)	
H5	−0.3207	0.6292	0.7634	0.041*	
C6	0.0389 (4)	0.5759 (3)	0.8297 (2)	0.0357 (5)	
H6	−0.0087	0.4799	0.8045	0.043*	
C7	0.0728 (4)	0.7525 (3)	0.6883 (2)	0.0393 (6)	
C8	0.2544 (5)	0.8648 (3)	0.7005 (3)	0.0524 (7)	
H8A	0.3755	0.8406	0.6514	0.079*	
H8B	0.3287	0.8765	0.7905	0.079*	
H8C	0.1774	0.9504	0.6675	0.079*	
C9	−0.0399 (6)	0.7197 (4)	0.5530 (3)	0.0654 (9)	
H9A	0.0828	0.6935	0.5056	0.098*	
H9B	−0.1246	0.8003	0.513	0.098*	
H9C	−0.1514	0.6438	0.5518	0.098*	
N1	0.4698 (3)	0.7562 (3)	1.2362 (2)	0.0498 (6)	
N2	0.4455 (3)	0.8362 (3)	1.3223 (2)	0.0464 (5)	
N3	0.4413 (5)	0.9168 (3)	1.3977 (3)	0.0654 (7)	
O1	0.1584 (3)	0.58380 (19)	0.95897 (16)	0.0426 (4)	
O2	−0.3347 (3)	0.5933 (2)	0.99674 (16)	0.0460 (4)	
H2A	−0.4782	0.6016	0.9625	0.069*	
O3	−0.0999 (3)	0.79118 (16)	0.76079 (15)	0.0386 (4)	
O4	0.1823 (3)	0.63255 (19)	0.74985 (18)	0.0497 (5)	
O5	−0.0267 (3)	0.74186 (18)	1.23341 (15)	0.0390 (4)	
H5A	−0.1747	0.7349	1.2142	0.059*	

### Atomic displacement parameters (Å^2^)

**Table:**

	*U*^11^	*U*^22^	*U*^33^	*U*^12^	*U*^13^	*U*^23^
C1	0.0349 (12)	0.0567 (16)	0.0369 (12)	0.0109 (11)	0.0075 (9)	0.0070 (11)
C2	0.0268 (11)	0.0403 (13)	0.0379 (12)	0.0022 (10)	0.0099 (9)	0.0035 (10)
C3	0.0271 (12)	0.0395 (12)	0.0340 (12)	0.0030 (9)	0.0105 (9)	−0.0009 (10)
C4	0.0246 (12)	0.0409 (13)	0.0376 (12)	0.0017 (9)	0.0086 (10)	0.0029 (10)
C5	0.0257 (12)	0.0402 (14)	0.0352 (12)	−0.0063 (9)	0.0027 (9)	−0.0005 (10)
C6	0.0323 (12)	0.0412 (13)	0.0349 (12)	−0.0046 (10)	0.0094 (9)	−0.0029 (10)
C7	0.0339 (13)	0.0491 (14)	0.0368 (13)	0.0019 (11)	0.0112 (10)	0.0015 (11)
C8	0.0435 (14)	0.0534 (18)	0.0639 (18)	−0.0069 (12)	0.0193 (13)	0.0006 (14)
C9	0.0598 (18)	0.094 (3)	0.0432 (16)	−0.0117 (17)	0.0118 (13)	−0.0108 (15)
N1	0.0259 (11)	0.0806 (18)	0.0433 (12)	−0.0012 (10)	0.0075 (9)	−0.0069 (12)
N2	0.0298 (10)	0.0663 (16)	0.0419 (13)	0.0060 (10)	0.0042 (9)	0.0052 (12)
N3	0.0656 (16)	0.0718 (17)	0.0578 (16)	0.0104 (13)	0.0099 (12)	−0.0061 (15)
O1	0.0278 (8)	0.0598 (11)	0.0404 (9)	0.0128 (8)	0.0071 (6)	−0.0047 (8)
O2	0.0220 (7)	0.0654 (11)	0.0521 (10)	−0.0030 (8)	0.0110 (7)	0.0101 (9)
O3	0.0346 (8)	0.0432 (10)	0.0396 (9)	0.0038 (7)	0.0110 (7)	0.0045 (7)
O4	0.0467 (10)	0.0532 (11)	0.0569 (11)	0.0106 (8)	0.0292 (8)	0.0106 (8)
O5	0.0269 (8)	0.0532 (11)	0.0389 (9)	0.0031 (7)	0.0113 (7)	−0.0032 (8)

### Geometric parameters (Å, º)

**Table:**

C1—N1	1.477 (4)	C6—O1	1.420 (3)
C1—C2	1.509 (3)	C6—H6	1
C1—H1A	0.99	C7—O3	1.427 (3)
C1—H1B	0.99	C7—O4	1.429 (3)
C2—O5	1.425 (3)	C7—C9	1.499 (4)
C2—C3	1.517 (3)	C7—C8	1.505 (4)
C2—H2	1	C8—H8A	0.98
C3—O1	1.451 (3)	C8—H8B	0.98
C3—C4	1.527 (3)	C8—H8C	0.98
C3—H3	1	C9—H9A	0.98
C4—O2	1.419 (3)	C9—H9B	0.98
C4—C5	1.522 (3)	C9—H9C	0.98
C4—H4	1	N1—N2	1.236 (3)
C5—O3	1.428 (3)	N2—N3	1.131 (4)
C5—C6	1.523 (3)	O2—H2A	0.84
C5—H5	1	O5—H5A	0.84
C6—O4	1.411 (3)		
			
N1—C1—C2	113.2 (2)	O4—C6—C5	105.36 (19)
N1—C1—H1A	108.9	O1—C6—C5	106.73 (17)
C2—C1—H1A	108.9	O4—C6—H6	111.6
N1—C1—H1B	108.9	O1—C6—H6	111.6
C2—C1—H1B	108.9	C5—C6—H6	111.6
H1A—C1—H1B	107.8	O3—C7—O4	105.03 (18)
O5—C2—C1	106.9 (2)	O3—C7—C9	111.3 (2)
O5—C2—C3	109.89 (18)	O4—C7—C9	109.7 (2)
C1—C2—C3	113.22 (17)	O3—C7—C8	107.8 (2)
O5—C2—H2	108.9	O4—C7—C8	108.8 (2)
C1—C2—H2	108.9	C9—C7—C8	113.7 (2)
C3—C2—H2	108.9	C7—C8—H8A	109.5
O1—C3—C2	107.18 (18)	C7—C8—H8B	109.5
O1—C3—C4	104.35 (18)	H8A—C8—H8B	109.5
C2—C3—C4	114.43 (17)	C7—C8—H8C	109.5
O1—C3—H3	110.2	H8A—C8—H8C	109.5
C2—C3—H3	110.2	H8B—C8—H8C	109.5
C4—C3—H3	110.2	C7—C9—H9A	109.5
O2—C4—C5	110.12 (19)	C7—C9—H9B	109.5
O2—C4—C3	108.23 (18)	H9A—C9—H9B	109.5
C5—C4—C3	101.95 (16)	C7—C9—H9C	109.5
O2—C4—H4	112	H9A—C9—H9C	109.5
C5—C4—H4	112	H9B—C9—H9C	109.5
C3—C4—H4	112	N2—N1—C1	115.41 (19)
O3—C5—C4	109.89 (18)	N3—N2—N1	173.0 (3)
O3—C5—C6	103.56 (16)	C6—O1—C3	110.24 (17)
C4—C5—C6	104.82 (18)	C4—O2—H2A	109.5
O3—C5—H5	112.6	C7—O3—C5	107.83 (18)
C4—C5—H5	112.6	C6—O4—C7	110.02 (17)
C6—C5—H5	112.6	C2—O5—H5A	109.5
O4—C6—O1	109.68 (18)		
			
N1—C1—C2—O5	−60.8 (2)	C4—C5—C6—O1	−15.1 (2)
N1—C1—C2—C3	60.3 (3)	C2—C1—N1—N2	81.8 (3)
O5—C2—C3—O1	−178.66 (18)	C1—N1—N2—N3	172 (2)
C1—C2—C3—O1	61.9 (3)	O4—C6—O1—C3	106.5 (2)
O5—C2—C3—C4	−63.5 (3)	C5—C6—O1—C3	−7.2 (2)
C1—C2—C3—C4	177.1 (2)	C2—C3—O1—C6	148.18 (18)
O1—C3—C4—O2	81.9 (2)	C4—C3—O1—C6	26.5 (2)
C2—C3—C4—O2	−34.9 (3)	O4—C7—O3—C5	−28.5 (2)
O1—C3—C4—C5	−34.2 (2)	C9—C7—O3—C5	90.2 (3)
C2—C3—C4—C5	−151.02 (19)	C8—C7—O3—C5	−144.4 (2)
O2—C4—C5—O3	164.46 (17)	C4—C5—O3—C7	139.25 (18)
C3—C4—C5—O3	−80.8 (2)	C6—C5—O3—C7	27.7 (2)
O2—C4—C5—C6	−84.8 (2)	O1—C6—O4—C7	−115.1 (2)
C3—C4—C5—C6	29.9 (2)	C5—C6—O4—C7	−0.5 (2)
O3—C5—C6—O4	−16.5 (2)	O3—C7—O4—C6	17.4 (2)
C4—C5—C6—O4	−131.67 (19)	C9—C7—O4—C6	−102.3 (2)
O3—C5—C6—O1	100.11 (19)	C8—C7—O4—C6	132.7 (2)

### Hydrogen-bond geometry (Å, º)

**Table:**

*D*—H···*A*	*D*—H	H···*A*	*D*···*A*	*D*—H···*A*
O2—H2*A*···O1^i^	0.84	2.09	2.871 (2)	154
O2—H2*A*···O4^i^	0.84	2.71	3.462 (2)	150
O5—H5*A*···N1^i^	0.84	2.12	2.910 (3)	157
C6—H6···O5^ii^	1.00	2.38	3.332 (3)	159

Symmetry codes: (i) *x*−1, *y*, *z*; (ii) −*x*, *y*−1/2, −*z*+2.

## References

[bb1] Altomare, A., Cascarano, G., Giacovazzo, C., Guagliardi, A., Burla, M. C., Polidori, G. & Camalli, M. (1994). *J. Appl. Cryst.* **27**, 435.

[bb2] Ashton, P. R., Ellwood, P., Staton, I. & Stoddart, J. F. (1991). *J. Org. Chem.* **56**, 7274–7280.

[bb3] Bilska-Markowska, M., Siodla, T., Patyk-Kaźmierczak, S., Katrusiak, A. & Koroniak, H. (2017). *New J. Chem.* **41**, 12631–12644.

[bb4] Chandran, S. K. & Nangia, A. (2006). *CrystEngComm*, **8**, 581–585.

[bb5] Chelouan, A., Bao, S., Friess, S., Herrera, A., Heinemann, F. W., Escalona, A., Grasruck, A. & Dorta, R. (2018). *Organometallics*, **37**, 3983–3992.

[bb6] Clarke, Z., Barnes, E., Prichard, K. L., Mares, L. J., Clegg, J. K., McCluskey, A., Houston, T. A. & Simone, M. I. (2018). *Acta Cryst.* E**74**, 862–867. 10.1107/S205698901800765X PMC6002814 29951246

[bb7] Farrugia, L. J. (2012). *J. Appl. Cryst.* **45**, 849–854.

[bb8] Flack, H. D. (1983). *Acta Cryst.* A**39**, 876–881.

[bb9] Fleet, G. W. J., Ramsden, N. G. & Witty, D. R. (1989). *Tetrahedron*, **45**, 327–336.

[bb10] Fujita, K. E., Ohta, K., Masunari, K., Obe, K. & Yamamura, H. (1992). *Tetrahedron Lett.* **33**, 5519–5520.

[bb11] Groom, C. R., Bruno, I. J., Lightfoot, M. P. & Ward, S. C. (2016). *Acta Cryst.* B**72**, 171–179. 10.1107/S2052520616003954 PMC4822653 27048719

[bb12] IUPAC (1996). *Pure Appl. Chem.* **68**, 1919–2008.

[bb13] Ivanovs, I., Bērziņa, S., Lugiņina, J., Belyakov, S. & Rjabovs, V. (2016). *Heterocycl. Commun.* **22**, 95–98.

[bb14] Johnson, L. L. & Houston, T. A. (2002). *Tetrahedron Lett.* **43**, 8905–8908.

[bb15] Johnson, W. S., Collins, J. C., Pappo, R., Rubin, M. B., Kropp, P. J., Johns, W. F., Pike, J. E. & Bartmann, W. (1963). *J. Am. Chem. Soc.* **85**, 1409–1430.

[bb16] Katritzky, A. R., Rachwal, S. & Hitchings, G. J. (1991). *Tetrahedron*, **47**, 2683–2732.

[bb17] Lee, J. C., Francis, S., Dutta, D., Gupta, V., Yang, Y., Zhu, J., Tash, J. S., Schönbrunn, E. & Georg, G. I. (2012). *J. Org. Chem.* **77**, 3082–3098. 10.1021/jo202054g PMC3431965 22432895

[bb18] Liu, Z., Yoshihara, A., Wormald, M. R., Jenkinson, S. F., Gibson, V., Izumori, K. & Fleet, G. W. J. (2014). *Org. Lett.* **16**, 5663–5665. 10.1021/ol502733x 25310515

[bb21] Mamat, C., Peppel, T. & Köckerling, M. (2012). *Crystals*, **2**, 105–109.

[bb22] Pradere, U., Roy, V., McBrayer, T. R., Schinazi, R. F. & Agrofoglio, L. A. (2008). *Tetrahedron*, **64**, 9044–9051. 10.1016/j.tet.2008.07.007 PMC8315236 34321698

[bb23] Prichard, K., Campkin, D., O’Brien, N., Kato, A., Fleet, G. W. J. & Simone, M. I. (2018). *Chem. Biol. Drug Des.* **92**, 1171–1197. 10.1111/cbdd.13182 29469975

[bb24] Rasmussen, T. S. & Jensen, H. H. (2011). *Carbohydr. Res.* **346**, 2855–2861. 10.1016/j.carres.2011.10.025 22088883

[bb25] Riediker, M., Hafner, A., Piantini, U., Rihs, G. & Togni, A. (1989). *Angew. Chem. Int. Ed. Engl.* **28**, 499–500.

[bb26] Rigaku (2015). *CrysAlis PRO*. Rigaku Corporation, Tokyo, Japan.

[bb27] Sayce, A. C., Alonzi, D. S., Killingbeck, S. S., Tyrrell, B. E., Hill, M. L., Caputo, A. T., Iwaki, R., Kinami, K., Ide, D., Kiappes, J. L., Beatty, P. R., Kato, A., Harris, E., Dwek, R. A., Miller, J. L. & Zitzmann, N. (2016). *PLoS Negl. Trop. Dis.* **10**, e0004524. 10.1371/journal.pntd.0004524 PMC4790851 26974655

[bb28] Šesták, S., Bella, M., Klunda, T., Gurská, S., Džubák, P., Wöls, F., Wilson, I. B. H., Sladek, V., Hajdúch, M., Poláková, M. & Kóňa, J. (2018). *ChemMedChem*, **13**, 373–383. 10.1002/cmdc.201700607 PMC6176901 29323461

[bb29] Sheldrick, G. M. (2008). *Acta Cryst.* A**64**, 112–122. 10.1107/S0108767307043930 18156677

[bb30] Simone, M. I., Soengas, R. G., Jenkinson, S. F., Evinson, E. L., Nash, R. J. & Fleet, G. W. J. (2012). *Tetrahedron Asymmetry*, **23**, 401–408.

[bb31] Steiner, A. J., Stütz, A. E., Tarling, C. A., Withers, S. G. & Wrodnigg, T. M. (2009). *Aust. J. Chem.* **62**, 553–557.

[bb32] Steiner, B., Langer, V. & Koóš, M. (2009). *Carbohydr. Res.* **344**, 2079–2082. 10.1016/j.carres.2009.06.031 19683708

[bb33] Tsuchiya, T., Miyake, T., Kageyama, S., Umezawa, S., Umezawa, H. & Takita, T. (1981). *Tetrahedron Lett.* **22**, 1413–1416.

[bb34] Tsuda, Y., Nishimura, M., Kobayashi, T., Sato, Y. & Kanemitsu, K. (1991). *Chem. Pharm. Bull.* **39**, 2883–2887.

[bb35] Wood, A., Prichard, K. L., Clarke, Z., Houston, T. A., Fleet, G. W. J. & Simone, M. I. (2018). *Eur. J. Org. Chem.* pp. 6812–6829.

[bb36] Woodhouse, S. D., Smith, C., Michelet, M., Branza-Nichita, N., Hussey, M., Dwek, R. A. & Zitzmann, N. (2008). *Antimicrob. Agents Chemother.* **52**, 1820–1828. 10.1128/AAC.01181-07 PMC2346636 18316522

[bb37] Yamamura, H. & Fujita, K. (1991). *Chem. Pharm. Bull.* **39**, 2505–2508.

[bb38] Zhang, Q., He, P., Zhou, G., Yu, K. & Liu, H. (2013). *Acta Cryst.* E**69**, o1386. 10.1107/S1600536813021351 PMC3884407 24427028

